# Normative Data for Novel Nominal Metaphors, Novel Similes, Literal, and Anomalous Utterances in Polish and English

**DOI:** 10.1007/s10936-020-09695-7

**Published:** 2020-03-07

**Authors:** Katarzyna Jankowiak

**Affiliations:** Faculty of English, Adam Mickiewicz University, Grunwaldzka 6, 60-780 Poznan, Poland

**Keywords:** Metaphors, Normative data, Novel nominal metaphors, Novel similes, Polish, English, Cloze probability, Meaningfulness, Familiarity, Metaphoricity, Comparison mechanisms

## Abstract

The two studies reported in the article provide normative measures for 120 novel nominal metaphors, 120 novel similes, 120 literal sentences, and 120 anomalous utterances in Polish (Study 1) and in English (Study 2). The presented set is ideally suited to addressing methodological requirements in research on metaphor processing. The critical (sentence-final) words of each utterance were controlled for in terms of their frequency per million, number of letters and syllables. For each condition in each language, the following variables are reported: cloze probability, meaningfulness, metaphoricity, and familiarity, whose results confirm that the sentences are well-matched. Consequently, the present paper provides materials that can be employed in order to test the new as well as existing theories of metaphor comprehension. The results obtained from the series of normative tests showed the same pattern in both studies, where the comparison structure present in similes (i.e., *A is like B*) facilitated novel metaphor comprehension, as compared to categorical statements (i.e., *A is B*). It therefore indicates that comparison mechanisms might be engaged in novel meaning construction irrespectively of language-specific syntactic rules.

## Introduction

In studies on language processing, the use of well-controlled stimuli is crucial, especially in research employing behavioral or neuroimaging methods, whose results are highly influenced by stimuli characteristics, including, but not limited to, word frequency, meaningfulness, and familiarity (Balota et al. 2006). It is therefore of a great importance to ensure that the stimuli used in such studies have been thoroughly normed for the variables that are important to control for, taking into account the specific research questions that are to be addressed in the experiment proper. Oftentimes, researchers employing quantitative research methods use a shared database of the stimuli that have already been appropriately normed so as to ensure consistency across different projects and laboratories, where the studies are conducted (Campbell and Raney 2016). The present paper provides a database on Polish (Study 1) and English (Study 2) novel nominal metaphors, novel similes, literal, and anomalous sentences that have all been normed on a number of factors in order to ensure that they can effectively be employed in further studies on novel metaphoric and literal language processing.

**Fig. 1:**
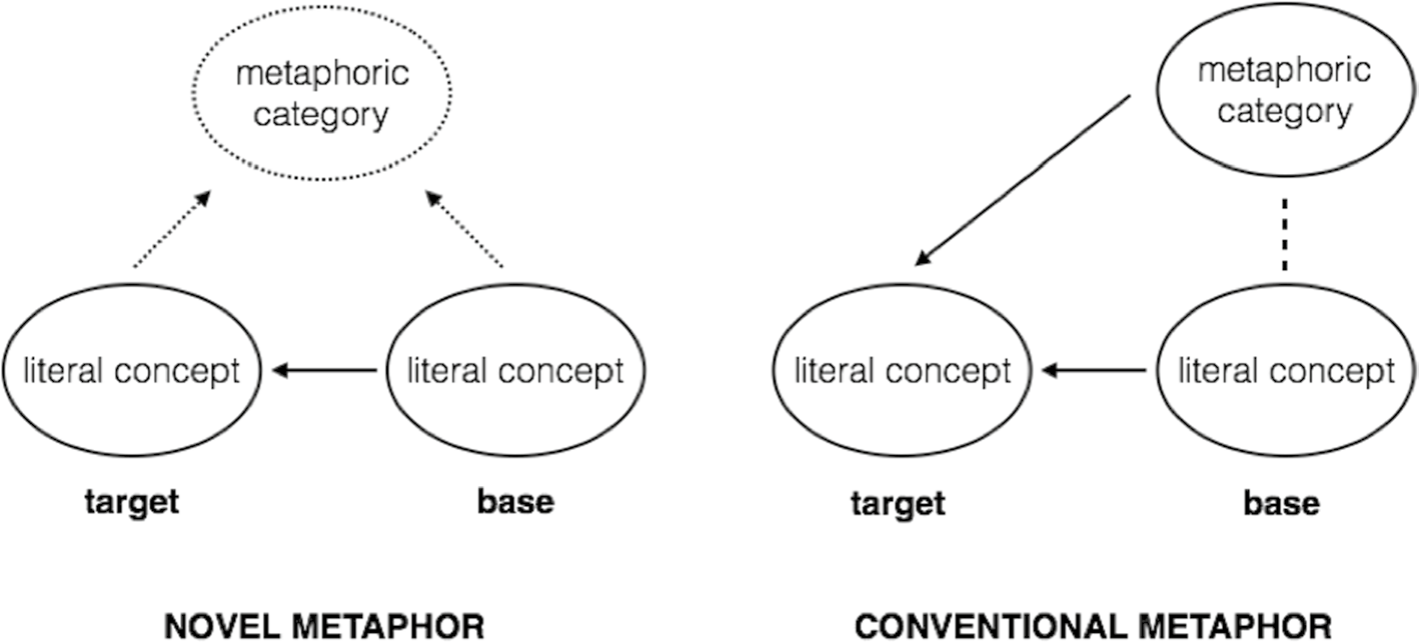
Novel and conventional metaphor comprehension according to the Career of Metaphor Model (after Bowdle and Gentner 1999: 92)

Metaphoric utterances, such as *That lawyer is a shark*, are defined as conveying meanings that do not refer to their literal sense (De Grauwe et al. 2010). Consequently, metaphor comprehension is assumed to require the process of cross-domain mapping, in which common features of two concepts (i.e., metaphor source—*shark*, and metaphor target—*lawyer*) need to be recognized and structurally aligned (Gibbs and Colston 2012; Bowdle and Gentner 2005). Importantly, as postulated within the Career of Metaphor Model (Bowdle and Gentner 2005), metaphor comprehension is highly modulated by how lexicalized (conventional) a metaphor source is, since a conventional metaphor source, as a result of its repeated use, has both a literal and metaphoric reference. Namely, a conventional metaphor such as *That lawyer is a shark* involves a source domain (*shark*) that is polysemous and can denote either a literal (i.e., a predator) or a nonliteral, metaphoric sense (i.e., someone who unscrupulously exploits others; Gentner and Bowdle 2001). This dual reference results in the fact that conventional metaphors might be comprehended as either comparisons or categorizations, with categorization mechanisms being more favorable, as they are faster and less cognitively intensive. In contrast, novel metaphors are not characterized by such a dual reference, as their source domains refer to domain-specific, literal concepts, as a result of which novel metaphoric utterances can be understood only by means of comparison. Consequently, while conventional (familiar) metaphors require meaning retrieval mechanisms, novel (unfamiliar) metaphoric utterances involve the processes of meaning construction that are based on comparison between the source and target domain. A distinction between novel and conventional metaphors, in line with the Career of Metaphor Model (Bowdle and Gentner 2005), is presented in Fig. 1.

The model therefore assumes that novel metaphoric meanings are easier to comprehend when presented as similes (*A is like B*) compared to nominal (categorical) sentences (*A is B*), due to the fact that the form of a simile automatically initiates comparison mechanisms that are engaged in novel metaphor processing. Such a hypothesis has previously been supported in a number of behavioral, electrophysiological, and neuroimaging studies (e.g., Bowdle and Gentner 2005; Shibata et al. 2012; Lai and Curran 2013), suggesting that the processing of novel similes is less cognitively taxing compared to novel nominal metaphors.

However, thus far little attention has been devoted to directly comparing novel metaphor comprehension across different languages. Consequently, the question whether the comparison structure facilitates novel meaning comprehension irrespectively of language-specific grammatical properties remains under-investigated. In order to provide valid insights into that notion, it seems crucial to compare languages in which a simile and categorical structure require a syntactically-different realization. For instance, in Polish and English, categorical statements differ in terms of their morpho-syntactic forms (i.e., Polish: *A to B*; English: *A is B*). Although in both languages, they include copular clauses, in English, a verbal copula *to be* needs to be explicitly stated, while in Polish, categorical sentences include a pronominal copula, which is dropped (Bondaruk 2014). On the other hand, using a verbal copula in Polish, which is a highly inflected language, would result in a sentence structure where a verbal copula *to be* (i.e., *A jest B*) marks the predicate for instrumental. In contrast, in the case of English categorical statements, a verbal copula *to be* (i.e., *A is B*) marks the predicate for nominative. It needs to be noted that since in Polish instrumental is much more structurally complex relative to nominative, metaphor source domains should be embedded in sentences where predicates are marked for nominative by using a pronominal copula (i.e., *A to B*). The two studies reported in the present article aim to show whether a comparison form present in similes facilitates novel metaphor comprehension in both Polish and English, with Polish nominal statements involving a pronominal copula, and English nominal statements involving a verbal copula.

Importantly, previous research into metaphor comprehension has indicated that, apart from metaphor conventionality, other factors modulating metaphoric meaning processing include the level of predictability, meaningfulness, and metaphoricity of the stimuli (De Grauwe et al. 2010; Jankowiak 2019). All of these variables might be measured by, for instance, employing rating Likert-type scales (Likert 1932), where participants rate utterances by means of selecting an appropriate numerical value on a predetermined scale (Gravetter and Forzano 2012). Likert-type scales have previously been used as a measure in conventionality (familiarity), meaningfulness, and metaphoricity ratings on metaphoric stimuli.

Familiarity is defined as the frequency of encountering an utterance (Gernsbacher 1984; Libben and Titone 2008; Tabossi et al. 2011; Nordmann et al. 2014), and has been shown to highly influence the process of word recognition to the point that it has been referred to as a strong predictor of the speed and accuracy of language processing (Connine et al. 1990; Ellis 2002). In research on metaphor processing, familiarity is understood in terms of meaning conventionality, with higher familiarity ratings for more conventional metaphors (Jankowiak et al. 2017). Additionally, the level of familiarity is interpreted as positively correlated with the subjective frequency of lexical items, as less familiar utterances are less frequently encountered in everyday language.

Next, the level of metaphoricity of an utterance provides answers to the question whether an expression, in order to make sense, needs to be interpreted literally or metaphorically. To this aim, metaphoricity ratings involve participants deciding how metaphorical or literal a given expression is. In studies on metaphor comprehension, metaphoricity ratings are aimed to confirm that novel metaphoric meanings are perceived as more metaphorical than conventional metaphors, both of which are more metaphorical than literal utterances (Arzouan et al. 2007; Yang et al. 2013).

Yet another variable that has been suggested to strongly modulate cognitive mechanisms engaged in metaphor comprehension is the predictability of an utterance (Sperber and Wilson 1986; Frisson and Pickering 2001; Katz and Ferretti 2001). The level of predictability is often tested by means of employing a cloze probability test, whose aim is to examine how much a context sentence suggests a to-be-inferred concept. Consequently, a cloze probability test is employed to investigate if the critical items are embedded in low or highly constraining contexts (Monzó and Calvo 2002). In such a test, participants are provided with utterances which are truncated before the final critical word (e.g., *She bought some apples and _______*). Respondents are asked to provide the first word that comes to their mind so that the sentence would be meaningful and grammatically correct (Bambini et al. 2013). It has been suggested that the processing of an upcoming lexical item is facilitated when presented in a predictable context compared to a non-predictable one (e.g., Kutas and Hillyard 1984; Zola 1984; Schwanenfluegel and Shoben 1985; Rayner and Pollatsek 1989). In metaphor processing, previous studies have revealed lower predictability for metaphoric than literal utterances, with novel metaphors eliciting lower results in cloze probability tests relative to conventional metaphors (e.g., Coulson and Van Petten 2002; Jankowiak et al. 2017).

All of the aforementioned variables influencing the process of metaphor comprehension have been tested in the two present studies, which were aimed to examine the level of predictability, meaningfulness, familiarity, and metaphoricity of Polish (Study 1) and English (Study 2) novel nominal metaphors, novel similes, literal, and anomalous sentences. By means of controlling the above-mentioned factors, the two studies provide valid insights into whether it is a comparison structure that facilitates novel meaning comprehension in Polish and English. As the two languages differ in the grammatical structures of categorical and comparison sentences, similar patterns of results observed in both Polish and English would indicate that the syntactic structure itself does not modulate complex semantic operations that are engaged in novel metaphor comprehension. Finally, the study aims to provide stimuli to serve as well-controlled materials perfectly suited to further examine a number of other different aspects of novel metaphoric meaning comprehension, and to test competing theories of how novel metaphors are constructed and processed in the human brain.

## Study 1: Polish Stimuli

### Method

#### Participants

Participants taking part in the surveys were all Polish native speakers, and were recruited from online social media, research mailing lists, and language forums. Participants spent less than 15 min to complete each survey. Importantly, raters who failed to complete the entire survey were removed from the analyses. Cloze probability tests were completed by 140 participants (*M*_*age*_ = 22.83, *SD* = 2.52; 123 females), meaningfulness ratings—by 132 participants (*M*_*age*_ = 21.8, *SD* = 2.4; 115 females), familiarity ratings—by 101 participants (*M*_*age*_ = 22.33, *SD* = 2.37; 80 females), and metaphoricity ratings—by 102 participants (*M*_*age*_ = 23.32; *SD* = 2.4, 87 females).

#### Materials and Design

Materials used in the ratings included 120 novel nominal metaphors (e.g., *Blizny to pamiętnik;* Eng: *Scars are a diary*), 120 novel similes (e.g., *Blizny są jak pamiętnik*; Eng: *Scars are like a diary*), 120 literal (e.g., *Ten egzemplarz to pamiętnik*; Eng: *This copy is a diary*), and 120 anomalous sentences (e.g., *Ten młot jest jak pamiętnik*; Eng: *This hammer is like a diary*). Each set shared the same sentence-final word, which was always a concrete noun. Novel nominal metaphors and novel similes shared the same target and source domain, and they differed only in their syntactic structure (i.e., *A to B*; Eng: *A is B* vs. *A jest jak B*; Eng: *A is like B*). Additionally, the critical words were controlled for in terms of their frequency per million (*M* = 4.68, *SD* = .45, range 2.47–4.7), number of syllables (*M* = 2.34, *SD* = .48, range 2–3), and number of letters (*M* = 6.57, *SD* = 1.45, range 4–11). Frequency values were calculated using the SUBTLEX-PL corpus (Mandera et al. 2014). The mean sentence lengths of the stimuli ranged from 3 to 5; novel nominal metaphors: *M* = 3.28, *SD* = .50, novel similes: *M* = 4.28, *SD* = .48, literal sentences: *M* = 4.00, *SD* = .18, and anomalous sentences: *M* = 3.71, *SD* = .57. The list of Polish stimuli is provided in Appendix 1.

#### Procedure

All of the normative studies were conducted using an online survey-development cloud-based software that enabled designing web-based surveys and collecting survey responses. For the normative tests, the materials were divided into four (meaningfulness ratings) or three (cloze probability tests, familiarity, and metaphoricity ratings) blocks so as to avoid the repetition of the critical word within one block. While meaningfulness ratings included all types of utterances (i.e., novel nominal metaphors, novel similes, literal, and anomalous sentences), normative tests on cloze probability, familiarity, and metaphoricity involved meaningful stimuli only (i.e., novel nominal metaphors, novel similes, and literal utterances).

Participants first gave their consent, after which they were provided with general instructions about the task. In all cases, the instructions were presented together with several examples and explanations. Each participant completed only one block, and rated the presented 120 sentences (in meaningfulness ratings) or 90 sentences (in cloze probability tests, familiarity, and metaphoricity ratings). All of the rating scales included 7-point Likert-type scales, i.e. meaningfulness ratings: 1—totally meaningless, 7—totally meaningful; familiarity ratings: 1—very rarely, 7—very frequently; metaphoricity ratings: 1—very literal, 7—very metaphorical). In cloze probability tests, raters were provided with the beginning of a sentence, and were asked to write a critical word (a noun) which first came to their mind, so that the whole sentence would be meaningful and syntactically correct. The order of stimuli presentation within each survey was randomized and counterbalanced across participants, with an equal number of stimuli per each condition presented to all participants.

For the normative studies with rating scales on stimuli meaningfulness, familiarity, and metaphoricity, analyses of variance (*ANOVA*s) were conducted, whose results are reported below. Significance values for pairwise comparisons were corrected for multiple comparisons using the Bonferroni correction. If Mauchly’s tests indicated that the assumption of sphericity was violated, the Greenhouse–Geisser correction was applied. In such cases, the original degrees of freedom are reported with the corrected *p* value.

### Results

To determine the reliability of the norming tests, intraclass correlation coefficients were calculated for all dimensions requiring a subjective rating. All measures indicated a high consistency across raters (Table 1).

**Table 1:** Study 1: Interclass correlation coefficients for the rating tasks

Dimension	Interclass correlation coefficient
Meaningfulness	.951
Familiarity	.983
Metaphoricity	.941

#### Cloze probability tests

Cloze probability tests were carried out with a view to ensuring that all of the critical (sentence-final) words were not expected due to the preceding context. Table 2 summarizes the results obtained from the cloze probability tests (reported as the percentage of participants who completed the presented sentence with a critical word) together with familiarity ratings and the correlation between the two variables.

**Table 2:** Study 1: Cloze probability and familiarity results, along with the correlation between the two variables, for novel nominal metaphors, novel similes, and literal sentences

	Novel nominal metaphors	Novel similes	Literal sentences
Cloze probability	*M* = .09%, *SD* = .51%	*M* = .38%, *SD* = 2.22%	*M* = 1.48%, *SD* = 4.63%
Familiarity	*M* = 1.8, *SD* = .89	*M* = 1.88, *SD* = .91	*M* = 2.51, *SD* = 1.24
Correlation between cloze probability and familiarity results	*r*(120) = .19, *p* = .036	*r*(120) = − .07, *p* > .05	*r*(120) = .17, *p* = .06

#### Meaningfulness ratings

To evaluate the meaningfulness of the sentences, raters assessed them on a scale from 1 (totally meaningless) to 7 (totally meaningful). The analysis showed a main effect of utterance type, *F*(3, 384) = 906.25, *p* < .001, ε = .774, η_p_^2^ = .876. Pairwise comparisons further revealed that literal sentences (*M* = 5.66, *SE* = .07) were rated as more meaningful than novel similes (*M* = 4.42, *SE* = .08), *p* < .001, novel similes were rated as more meaningful than novel nominal metaphors (*M* = 3.87, *SE* = .08), *p* < .001, and novel nominal metaphors were assessed as more meaningful compared to anomalous utterances (*M* = 1.80, *SE* = .06), *p* < .001.

#### Familiarity ratings

In order to examine the familiarity of the stimuli, raters decided how often they encountered the presented novel nominal metaphors, novel similes, and literal sentences on a scale from 1 (very rarely) to 7 (very frequently). The obtained results revealed a main effect of sentence type, *F*(2, 196) = 45.94, *p* < .001, ε = .562, η_p_^2^ = .319. Pairwise comparisons confirmed that novel nominal metaphors (*M* = 1.80, *SE* = .09) were less familiar than both novel similes (*M* = 1.88, *SE* = .09), *p* = .014, and literal sentences (*M* = 2.51, *SE* = .13), *p* < .001. Furthermore, novel similes were less familiar than literal utterances, *p* < .001.

#### Metaphoricity ratings

**Fig. 2:**
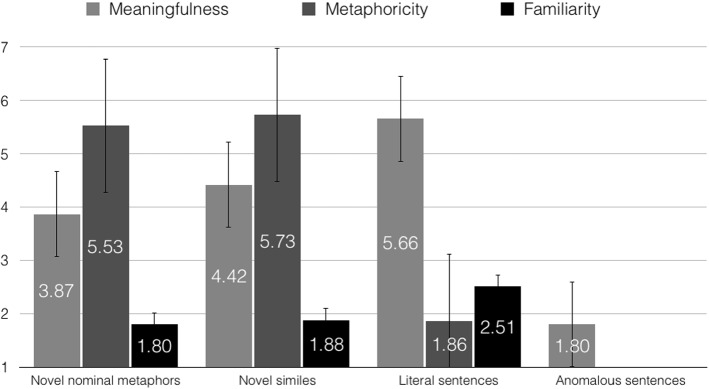
Study 1: Meaningfulness, metaphoricity, and familiarity ratings for novel nominal metaphors, novel similes, literal, and anomalous sentences

In order to assess the metaphoricity of the stimuli, raters decided how metaphorical or literal novel nominal metaphors, novel similes, and literal sentences were on a scale from 1 (very literal) to 7 (very metaphorical). The analysis showed a main effect of sentence type, *F*(2, 198) = 902.18, *p* < .001, ε = .658, η_p_^2^ = .901. Pairwise comparisons further showed that novel similes (*M* = 5.73, *SE* = .08) were rated as more metaphorical than novel nominal metaphors (*M* = 5.53, *SE* = .08), *p* = .001, as well as than literal sentences (*M* = 1.86, *SE* = .07), *p* < .001. Additionally, novel nominal metaphors were rated as more metaphorical than literal utterances, *p* < .001. Figure 2 presents meaningfulness, metaphoricity, and familiarity ratings for the materials.

The overall means obtained from the normative tests confirmed desired differences between the conditions. Table 3 provides interscale correlations between stimuli dimensions, and Table 4 presents the summary of stimuli characteristics by sentence type.

**Table 3:** Study 1: Interscale correlations between stimuli dimensions

	Cloze probability	Meaningfulness	Familiarity	Metaphoricity
*Novel nominal metaphors*				
Cloze probability	1	− .006 *p* > .05	.192* *p* = .036	− .102 *p* > .05
Meaningfulness	− .006 *p* > .05	1	.667** *p* < .005	.102 *p* > .05
Familiarity	.192* *p* = .036	.667** *p* < .005	1	.198* *p* = .03
Metaphoricity	− .102 *p* > .05	.102 *p* > .05	.198* *p* = .03	1
*Novel similes*				
Cloze probability	1	.083 *p* > .05	− .071 *p* > .05	− .287** *p* < .001
Meaningfulness	.083 *p* > .05	1	.000 *p* > .05	− .009 *p* > .05
Familiarity	− .071 *p* > .05	.000 *p* > .005	1	− .109 *p* > .05
Metaphoricity	− .287** *p* < .001	− .009 *p* > .05	− .109 *p* > .05	1
*Literal sentences*				
Cloze probability	1	.130 *p* > .05	.170* *p* = .06	− .094 *p* > .05
Meaningfulness	.130 *p* > .05	1	.654** *p* < .001	− .579** *p* < .001
Familiarity	.170* *p* = .06	.654** *p* < .001	1	− .454** *p* < .001
Metaphoricity	− .094 *p* > .05	− .579** *p* < .001	− .454** *p* < .001	1

**Table 4:** Study 1: Summary of stimuli characteristics by sentence type

	Novel nominal metaphors			Novel similes			Literal sentences			Anomalous sentences		
	*M*	*SD*	Range	*M*	*SD*	Range	*M*	*SD*	Range	*M*	*SD*	Range
Number of words per sentence	3.29	.50	3–5	4.28	.48	4–6	4.00	.18	3–5	3.71	.57	3–5
Meaningfulness ratings	3.83	.79	2.37–6.14	4.36	.82	2.29–5.91	5.56	.65	3.57–6.63	1.82	.31	1.28–2.86
Familiarity ratings	1.84	.44	1.2–3.26	1.92	.49	1.17–3.51	2.51	.53	1.43–4.57	n/a		
Metaphoricity ratings	5.51	.47	4–6.28	5.69	.36	4.68–6.28	1.93	.46	1.34–4.74	n/a		

## Study 2: English Stimuli

### Method

#### Participants

Participants taking part in the web-based surveys were all English native speakers, and were recruited from online social media, research mailing lists, and language forums. Participants spent less than 15 min to complete each survey. Importantly, raters who failed to complete the entire survey were removed from the analyses. Cloze probability tests were completed by 152 participants (*M*_*age*_ = 26.31, *SD* = 9.99; 78 females), meaningfulness ratings—by 119 participants (*M*_*age*_ = 24.28, *SD* = 8.03; 62 females), familiarity ratings—by 87 participants (*M*_*age*_ = 26.61, *SD* = 10.2; 40 females), and metaphoricity ratings—by 87 participants (*M*_*age*_ = 25.96, *SD* = 9.02; 44 females).

#### Materials and Design

Materials used in the ratings included 120 novel nominal metaphors (e.g., *Viruses are travellers*), 120 novel similes (e.g., *Viruses are like travellers*), 120 literal (e.g., *These people are travellers*), and 120 anomalous sentences (e.g., *Tables are travellers*). Similarly to the stimuli tested in Study 1, each set shared the same sentence-final word, which was always a concrete noun. Novel nominal metaphors and novel similes shared the same target and source domain, and they differed only in their syntactic structure (i.e., *A is B* vs. *A is like B*). The critical words were controlled for in terms of their frequency per million (*M* = 3.93, *SD* = .56, range 2.47–4.95), number of syllables (*M* = 2.19, *SD* = .39, range 2–3), and number of letters (*M* = 6.87, *SD* = 1.24, range 5–10). Frequency values were calculated using the SUBTLEX-UK corpus (van Heuven et al. 2014). The mean sentence lengths of the stimuli ranged from 3 to 7; novel nominal metaphors: *M* = 4.12, *SD* = .82, novel similes: *M* = 5.12, *SD* = .83, literal sentences: *M* = 4.82, *SD* = .66, and anomalous sentences: *M* = 4.86, *SD* = .94. The list of English stimuli is provided in Appendix 2.

#### Procedure

The procedures applied in the normative tests on English stimuli were the same as those used in Study 1.

### Results

To determine the reliability of the norming tests, intraclass correlation coefficients were calculated for all dimensions requiring a subjective rating. All measures indicated high consistency across raters (Table 5).

**Table 5:** Study 2: Interclass correlation coefficients for the rating tasks

Dimension	Interclass correlation coefficient
Meaningfulness	.910
Familiarity	.946
Metaphoricity	.890

#### Cloze Probability Tests

Cloze probability tests were carried out with a view to ensuring that all of the critical (sentence-final) words were not expected due to the preceding context. Table 6 summarizes the results obtained from the cloze probability tests (reported as the percentage of participants who completed the presented sentence with a critical word), together with familiarity ratings and the correlation between the two variables.

**Table 6:** Study 2: Cloze probability and familiarity results, along with the correlation between the two variables, for novel nominal metaphors, novel similes, and literal sentences

	Novel nominal metaphors	Novel similes	Literal sentences
Cloze probability	*M* = .18%, *SD* = .84%	*M* = .86%, *SD* = 3.60%	*M* = 3.46%, *SD* = 7.63%
Familiarity	*M* = 2, *SD* = .56	*M* = 2.13, *SD* = .64	*M* = 2.92, *SD* = 1.03
Correlation between cloze probability and familiarity results	*r*(120) = .19, *p* = .036	*r*(120) = .35, *p* < .001	*r*(120) = .1, *p* > .05

#### Meaningfulness Ratings

To evaluate the meaningfulness of the sentences, raters assessed them on a scale from 1 (totally meaningless) to 7 (totally meaningful). The analysis showed a main effect of utterance type, *F*(3, 345) = 2026.18, *p* < .001, ε = .872, η_p_^2^ = .946. Pairwise comparisons further revealed that literal sentences (*M* = 5.92, *SE* = .05) were rated as more meaningful than novel similes (*M* = 4.98, *SE* = .05), *p* < .001, novel similes were rated as more meaningful than novel nominal metaphors (*M* = 4.50, *SE* = .05), *p* < .001, and novel nominal metaphors were assessed as more meaningful compared to anomalous utterances (*M* = 1.85, *SE* = .05), *p* < .001.

#### Familiarity Ratings

In order to examine the familiarity of the stimuli, raters decided how often they encountered the presented novel nominal metaphors, novel similes, and literal sentences on a scale from 1 (very rarely) to 7 (very frequently). The obtained results revealed a main effect of sentence type, *F*(2, 168) = 159.86, *p* < .001, ε = .661, η_p_^2^ = .656. Pairwise comparisons confirmed that novel nominal metaphors (*M* = 2.00, *SE* = .06) were less familiar than both novel similes (*M* = 2.13, *SE* = .07), *p* < .001, and literal sentences (*M* = 2.92, *SE* = .10), *p* < .001. Furthermore, novel similes were less familiar than literal utterances, *p* < .001.

#### Metaphoricity Ratings

**Fig. 3:**
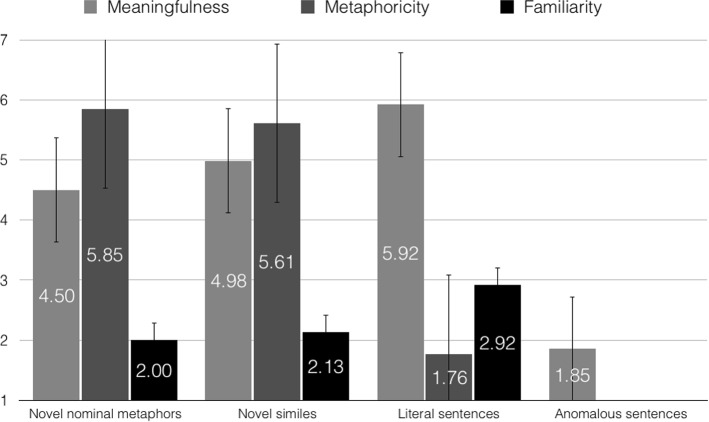
Study 2: Meaningfulness, metaphoricity, and familiarity ratings for novel nominal metaphors, novel similes, literal, and anomalous sentences

In order to assess the metaphoricity of the stimuli, raters decided how metaphorical or literal novel nominal metaphors, novel similes, and literal sentences were on a scale from 1 (very literal) to 7 (very metaphorical). The analysis showed a main effect of sentence type, *F*(2, 168) = 2466.83, *p* < .001, ε = .847, η_p_^2^ = .967. Pairwise comparisons further showed that novel nominal metaphors (*M* = 5.85, *SE* = .04) were rated as more metaphorical than novel similes (*M* = 5.61, *SE* = .07), *p* = .001, as well as than literal sentences (*M* = 1.76, *SE* = .03), *p* < .001. Additionally, novel similes were rated as more metaphorical than literal utterances, *p* < .001. Figure 3 presents meaningfulness, metaphoricity, and familiarity ratings for the materials.

The overall means obtained from the normative tests confirmed desired differences between the conditions. Table 7 provides interscale correlations between stimuli dimensions, and Table 8 presents the summary of stimuli characteristics by sentence type.

**Table 7:** Study 2: Interscale correlations between stimuli dimensions

	Cloze probability	Meaningfulness	Familiarity	Metaphoricity
*Novel nominal metaphors*				
Cloze probability	1	.040 *p* > .05	.023 *p* > .05	.014 *p* > .05
Meaningfulness	.040 *p* > .05	1	.760** *p* < .001	− .317** *p* < .001
Familiarity	.023 *p* > .05	.760** *p* < .001	1	− .359** *p* < .001
Metaphoricity	.014 *p* > .05	− .317** *p* < .001	− .359** *p* < .001	1
*Novel similes*				
Cloze probability	1	− .003 *p* > .05	.001 *p* > .05	.072 *p* > .05
Meaningfulness	− .003 *p* > .05	1	.760** *p* < .001	− .264** *p* < .001
Familiarity	.001 *p* > .05	.760** *p* < .001	1	− .297** *p* < .001
Metaphoricity	.072 *p* > .05	− .264** *p* < .001	− .297** *p* < .001	1
*Literal sentences*				
Cloze probability	1	.120 *p* > .05	.056 *p* > .05	− .004 *p* > .05
Meaningfulness	.120 *p* > .05	1	.714** *p* < .001	− .475 *p* < .001
Familiarity	.056 *p* > .05	.714** *p* < .001	1	− .475 *p* < .001
Metaphoricity	− .004 *p* > .05	− .475 *p* < .001	− .475 *p* < .001	1

**Table 8:** Study 2: Summary of stimuli characteristics by sentence type

	Novel nominal metaphors			Novel similes			Literal sentences			Anomalous sentences		
	*M*	*SD*	Range	*M*	*SD*	Range	*M*	*SD*	Range	*M*	*SD*	Range
Number of words per sentence	4.12	.82	3–6	5.12	.83	4–7	4.82	.66	4–7	4.86	.94	3–6
Meaningfulness ratings	4.50	1.03	2.48–6.44	4.98	.89	3.03–6.74	5.92	.72	3.59–6.97	1.85	.36	1.23–2.52
Familiarity ratings	2.00	.78	1.03–4.93	2.13	.74	1.10–4.67	2.92	.86	1.40–5.37	n/a		
Metaphoricity ratings	5.85	.80	1.76–6.76	5.61	.55	3.28–6.34	1.76	.68	1.00–4.17	n/a		

## Correlation Analyses Between Study 1 and Study 2

**Fig. 4:**
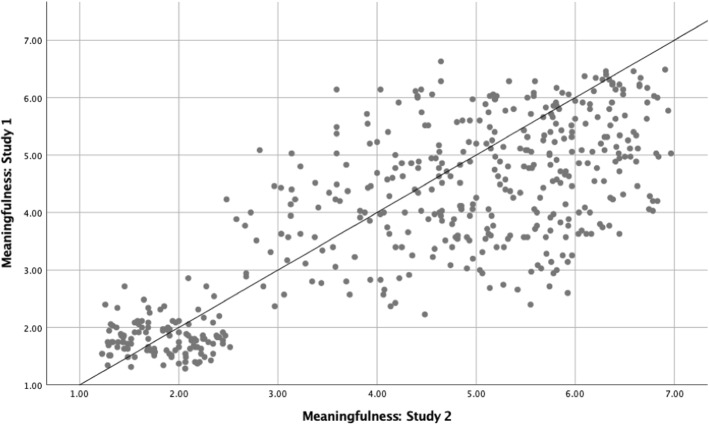
Scatterplots presenting the correlation analyses between meaningfulness ratings for Study 1 and Study 2

**Fig. 5:**
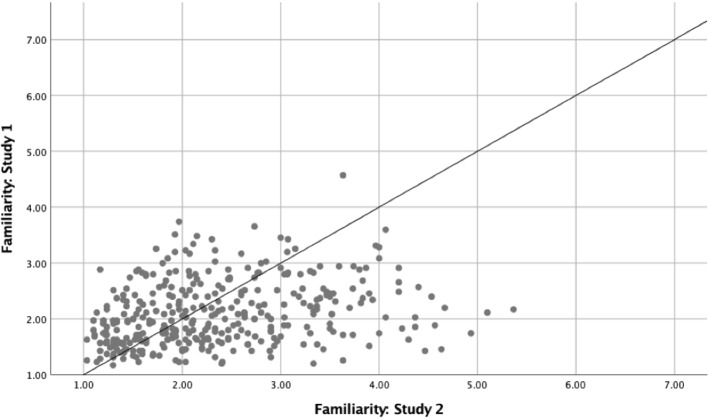
Scatterplots presenting the correlation analyses between familiarity ratings for Study 1 and Study 2

**Fig. 6:**
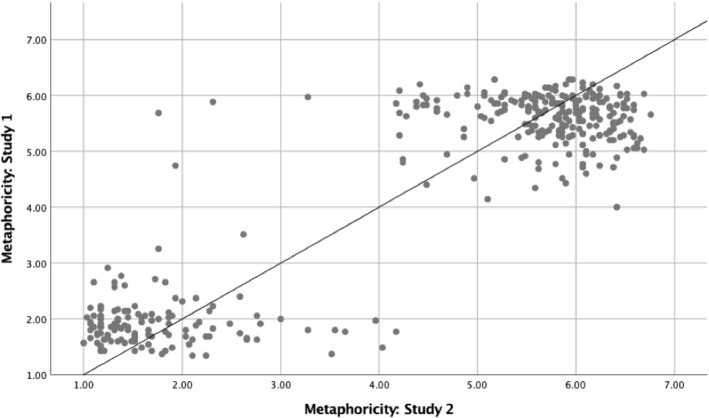
Scatterplots presenting the correlation analyses between metaphoricity ratings for Study 1 and Study 2

The correlation analyses were carried out for Study 1 and Study 2 on the meaningfulness, familiarity, and metaphoricity ratings, and were conducted on averaged values for all novel similes, novel nominal metaphors, literal, and anomalous sentences. The results showed a strong positive correlation between in the two studies on meaningfulness ratings (*r*(478) = .79, *p* < .001), familiarity ratings (*r*(358) = .28, *p* < .001), and metaphoricity ratings (*r*(358) = .90, *p* < .001). Figures 4, 5, and 6 show scatterplots representing the correlations between the two studies on meaningfulness, familiarity, and metaphoricity, respectively.

## Discussion and Conclusions

The present article was aimed to provide normative data for a set of Polish (Study 1) and English (Study 2) novel nominal metaphors, novel similes, literal, and anomalous sentences, and to show whether differences in the syntactic structures of the two languages modulate comparison and categorization mechanisms engaged in novel metaphor comprehension.

The obtained results showed that novel similes elicited higher meaningfulness ratings compared to novel nominal metaphors, which is in line with the Career of Metaphor Model (Bowdle and Gentner 2005), and indicates that comparison processes initiated by similes facilitate novel meaning comprehension, and consequently, a form of a simile might ease novel metaphor construction. Under this view, novel metaphoric meanings need to undergo the process of comparison and alignment between the target domain and the literal meaning of the source domain (Bowdle and Gentner 1999; Zharikov and Gentner 2002). Such results are in line with a reaction time (RT) study conducted by Bowdle and Gentner (2005), who observed faster RTs for novel comparisons than novel categorizations, as well as in accordance with an fMRI (functional Magnetic Resonance Imaging) study conducted by Shibata et al. (2012), who found extended brain activation in the right inferior frontal gyrus in response to novel nominal metaphors relative to novel similes.

The observed results might also be interpreted as in line with the Relevance Theory (Sperber and Wilson 1986), which views meaning conventionality as a modulating factor in metaphor vs. simile comprehension, and assumes different explicatures that are communicated by the two types of utterances. Namely, novel creative metaphorical meanings are more likely to be conveyed via similes, which are postulated to require lexically encoded concepts. Conventionalized meanings, in contrast, are usually understood in terms of ad hoc concepts, whose meanings can be implied by typical connotations of metaphor source concept (Carston and Wearing 2011; Jankowiak 2019).

A preference for similes in communicating novel meanings is also in line with the Semantic Space Model (Utsumi 2011), which is based on the Predication Model (Kintsch 2001). In the Semantic Space Model, the meaning of each lexical item takes the form of a semantic space based on word occurrence. Based on this, a semantic similarity between two lexical items can be computed by comparing their respective semantic spaces. In the simulation experiment, Utsumi (2011) showed that categorization processes facilitate conventionalized meaning comprehension, while semantically diverse entities (i.e., novel metaphors) are easier to be interpreted as comparisons.

Importantly, the present results showed the same pattern of results in both Polish and English, therefore indicating that mechanisms involved in novel metaphor processing are similar even in languages with different syntactic rules governing the structure of similes and categorical statements. Thus, though there are differences in the morpho-syntactic forms of Polish and English categorical statements (i.e., Polish: *A to B*; English: *A is B*), with a verbal copula *to be* explicitly stated in English, and a pronominal copula dropped in Polish (Bondaruk 2014), the cognitive mechanisms engaged in arriving at semantically complex, novel metaphoric meanings seem to be irrespective of such language-specific differences. This in turn suggests that mental operations involved in semantic analyses and meaning integration might be superior to syntactic analyses, probably due to extended semantic mechanisms that are required when comprehending unfamiliar, poetic meanings. Further research is, nonetheless, needed in order to examine whether similar patterns of results would be observed in behavioral or electrophysiological studies employing such a between-language design to investigate comparison mechanisms in novel metaphor comprehension.

In addition, meaningfulness ratings for the two studies showed that literal utterances were perceived as most meaningful, followed by novel similes, novel nominal metaphors, and finally anomalous sentences. Importantly, novel metaphors, being very poetic and rarely used in everyday language, still elicited higher meaningfulness ratings as compared to anomalous utterances, thus confirming that the raters were able to arrive at a nonliteral interpretation of novel metaphoric meanings.

The stimuli presented in the present paper have been thoroughly examined in terms of their level of predictability, meaningfulness, familiarity (conventionality), and metaphoricity. Additionally, the critical (final) words of each sentence were selected so that they were all matched on their frequency, concreteness, number of letters and syllables. This is of special importance for any behavioral and electrophysiological research, where dependent variables (e.g., reaction times, event-related brain potentials) are time-locked to the presentation of the sentence-final word. As a result, it is assured that the observed differences in the results elicited in response to different conditions are dependent only on the category of a sentence (i.e., metaphorical, literal, or anomalous), as other lexico-semantic variables influencing language processing were highly controlled for.

Importantly, previous studies on metaphor comprehension have rarely tested stimuli in four separate normative tests (i.e., cloze probability, meaningfulness, metaphoricity, and familiarity), and they most often involved familiarity and/or interpretability ratings, or were limited to controlling only the critical items in terms of their frequency and number of letters. Additionally, previous research has rarely employed 7-point Likert scales, while using such a scale has been proven advantageous, as raters often seem to avoid extreme categories (e.g., 1 or 7), and therefore a scale with fewer than 5 categories would likely provide a too limited number of responses to select from. Furthermore, raters might find it relatively difficult to discriminate between too many categories, and as a result, they could blend the them when provided with more than 9 categories to choose from (Gravetter and Forzano 2012).

The aim of the two present studies was to provide a database of Polish and English novel nominal metaphors, novel similes, literal, and anomalous sentences with variables that have been previously shown to impact the processing of such utterances (i.e., predictability, meaningfulness, familiarity, metaphoricity). The same patterns of results observed in both Polish and English suggest that, although the two languages differ in their grammatical rules to a great extent, the syntactic structures themselves seem not to modulate comparison and categorization mechanisms engaged in novel metaphor comprehension, therefore suggesting that novel simile comprehension is facilitated by a comparison form, irrespectively of language. The large number of items and normed factors influencing language processing that are provided in the two present studies maximize the stimuli flexibility, as they can be effectively employed to suit a number of different research questions, populations, tasks, and designs. The final set of stimuli was selected so that novel similes, novel nominal metaphors, literal, and anomalous sentences are matched for sentence length, meaningfulness, familiarity, metaphoricity, and cloze probability, and the critical words are additionally matched for frequency, number of letters and syllables. The present database will hopefully provide a useful tool to anyone researching the processing and comprehension of metaphoric meanings in Polish and English, in both the monolingual and bilingual populations.

## Appendix 1

See Table 9.

**Table 9:** Polish stimuli

	Novel nominal metaphors	Novel similes	Literal sentences	Anomalous sentences
1	Życie to parkiet	Życie jest jak parkiet	Ta nawierzchnia to parkiet	Cukierek jest jak parkiet
2	Twarz to plakat	Twarz jest jak plakat	Ten arkusz to plakat	Mąka jest jak plakat
3	Błąd to przepaść	Błąd jest jak przepaść	Ta szczelina to przepaść	Manekin jest jak przepaść
4	Zatarg to płomień	Zatarg jest jak płomień	To światło to płomień	Klucz to płomień
5	Miłość to klasztor	Miłość jest jak klasztor	Ten zabytek to klasztor	Dywan jest jak klasztor
6	Natura to apteka	Natura jest jak apteka	Ten sklep to apteka	Czapka to apteka
7	Uczucia to oddech	Uczucia są jak oddech	Ten proces to oddech	Rękawice są jak oddech
8	Uczelnia to królestwo	Uczelnia jest jak królestwo	Szwecja to królestwo	Cyrkiel jest jak królestwo
9	Pamięć to torba	Pamięć jest jak torba	To opakowanie to torba	Ekran to torba
10	Przeszłość to plama	Przeszłość jest jak plama	Ten ślad to plama	Hamulec to plama
11	Uroda to korona	Uroda jest jak korona	Ta ozdoba to korona	Linijka jest jak korona
12	Talent to winda	Talent jest jak winda	To pomieszczenie to winda	Kałuża jest jak winda
13	Skóra to koszula	Skóra jest jak koszula	To okrycie to koszula	Wiatrak to koszula
14	Mózg to szafa	Mózg jest jak szafa	Ten mebel to szafa	Gazeta to szafa
15	Obrączka to łańcuch	Obrączka jest jak łańcuch	Ta linia to łańcuch	Kredka to łańcuch
16	Instynkt to władca	Instynkt jest jak władca	Ten rycerz to władca	Śliwka jest jak władca
17	Człowiek to kropla	Człowiek jest jak kropla	Ta resztka to kropla	Brama jest jak kropla
18	Życiorys to ulotka	Życiorys jest jak ulotka	Ten druczek to ulotka	Lakier jest jak ulotka
19	Tradycja to skała	Tradycja jest jak skała	Ta przeszkoda to skała	Śniadanie to skała
20	Nieśmiałość to korek	Nieśmiałość jest jak korek	To tworzywo to korek	Klamka to korek
21	Osobowość to krajobraz	Osobowość jest jak krajobraz	Ten malunek to krajobraz	Włócznia jest jak krajobraz
22	Brzuch to worek	Brzuch jest jak worek	Ten plastik to worek	Szlaban jest jak worek
23	Sumienie to dzwonek	Sumienie jest jak dzwonek	Ten instrument to dzwonek	Chleb jest jak dzwonek
24	Rozsądek to adwokat	Rozsądek jest jak adwokat	Ten zawód to adwokat	Kierownica to adwokat
25	Istnienie to świeca	Istnienie jest jak świeca	To oświetlenie to świeca	Rower jest jak świeca
26	Kościół to schronisko	Kościół jest jak schronisko	Ten dom to schronisko	Grabie to schronisko
27	Makijaż to tarcza	Makijaż jest jak tarcza	To uzbrojenie to tarcza	Sałatka jest jak tarcza
28	Potomek to lustro	Potomek jest jak lustro	Ta powierzchnia to lustro	Gałąź jest jak lustro
29	Rozterki to burza	Rozterki są jak burza	Ten odgłos to burza	Wazon to burza
30	Starość to kronika	Starość jest jak kronika	Ta książka to kronika	Spinka jest jak kronika
31	Ten związek to wózek	Ten związek jest jak wózek	Ten pojazd to wózek	Kabel jest jak wózek
32	Prawo to mundur	Prawo jest jak mundur	Ten strój to mundur	Piekarnik jest jak mundur
33	Ciało to koperta	Ciało jest jak koperta	Ten papier to koperta	Woda jest jak koperta
34	Serce to zegar	Serce jest jak zegar	Ten przyrząd to zegar	Nożyczki są jak zegar
35	Maniery to garnitur	Maniery są jak garnitur	To ubranie to garnitur	Chodnik to garnitur
36	Jej garderoba to kaplica	Jej garderoba jest jak kaplica	Ten budynek to kaplica	Piasek to kaplica
37	Rozpacz to powódź	Rozpacz jest jak powódź	To zjawisko to powódź	Strzelba jest jak powódź
38	Jego mięśnie to ciasto	Jego mięśnie są jak ciasto	Ten deser to ciasto	Bluzka jest jak ciasto
39	Rodzeństwo to wagon	Rodzeństwo jest jak wagon	Ten magazyn to wagon	Drzwi są jak wagon
40	Rozwód to pogrzeb	Rozwód jest jak pogrzeb	Ten rytuał to pogrzeb	Stopa jest jak pogrzeb
41	Jej figura to cegła	Jej figura jest jak cegła	Ten materiał to cegła	Szklanka jest jak cegła
42	Piec to żołądek	Piec jest jak żołądek	Ten organ to żołądek	Robak jest jak żołądek
43	Uśmiech to sztandar	Uśmiech jest jak sztandar	Ten znak to sztandar	Łyżka to sztandar
44	Ten docinek to pocisk	Ten docinek jest jak pocisk	Ta kulka to pocisk	Okno jest jak pocisk
45	Wiedza to laska	Wiedza jest jak laska	Ten rekwizyt to laska	Kapsel jest jak laska
46	Emerytura to ławka	Emerytura jest jak ławka	Ta deska to ławka	Widły są jak ławka
47	Młodość to zając	Młodość jest jak zając	To zwierzę to zając	Ręcznik jest jak zając
48	Zakochanie to cukier	Zakochanie jest jak cukier	Ten towar to cukier	Armia to cukier
49	Kac to pustynia	Kac jest jak pustynia	Ten obszar to pustynia	Pomadka jest jak pustynia
50	Opinia to flaga	Opinia jest jak flaga	Ten symbol to flaga	Taczka jest jak flaga
51	Autostrada to taśma	Autostrada jest jak taśma	To wykończenie to taśma	Siodło to taśma
52	Pożyczka to karetka	Pożyczka jest jak karetka	Ten samochód to karetka	Ramka to karetka
53	Akcent to pamiątka	Akcent jest jak pamiątka	Ten wisiorek to pamiątka	Gardło to pamiątka
54	Kłopoty to chmura	Kłopoty są jak chmura	Ten widok to chmura	Szynka jest jak chmura
55	Kołyska to gniazdo	Kołyska jest jak gniazdo	Ta konstrukcja to gniazdo	Zakładka to gniazdo
56	Krytyka to karabin	Krytyka jest jak karabin	Ta broń to karabin	Tama to karabin
57	Ta widownia to zwłoki	Ta widownia jest jak zwłoki	Ten dowód to zwłoki	Dzida to zwłoki
58	Jej młodsza siostra to mucha	Jej młodsza siostra jest jak mucha	To stworzenie to mucha	Łopata jest jak mucha
59	Ten grubas to jajko	Ten grubas jest jak jajko	Ten posiłek to jajko	Puder to jajko
60	Muzyka to zabawka	Muzyka jest jak zabawka	Ten prezent to zabawka	Tajfun to zabawka
61	Ten naród to stado	Ten naród jest jak stado	Ta gromada to stado	Zapałka jest jak stado
62	Psycholog to kapłan	Psycholog jest jak kapłan	Ten mężczyzna to kapłan	Przewód to kapłan
63	Ta fryzura to rzeźba	Ta fryzura jest jak rzeźba	Ta dekoracja to rzeźba	Walizka to rzeźba
64	Ta dziewczyna to pióro	Ta dziewczyna jest jak pióro	Ten przedmiot to pióro	Kurtka to pióro
65	Intelekt to mięsień	Intelekt jest jak mięsień	To wybrzuszenie to mięsień	Beczka to mięsień
66	Słowo to farba	Słowo jest jak farba	Ta ciecz to farba	Pierścień jest jak farba
67	Plotka to iskra	Plotka jest jak iskra	Ten błysk to iskra	Gałka to iskra
68	Rozum to świątynia	Rozum jest jak świątynia	Ta budowla to świątynia	Podeszwa jest jak świątynia
69	Jego głowa to ziemniak	Jego głowa jest jak ziemniak	To warzywo to ziemniak	Balsam jest jak ziemniak
70	Wiara to przewodnik	Wiara jest jak przewodnik	Ta osoba to przewodnik	Słoik jest jak przewodnik
71	Pies to badacz	Pies jest jak badacz	Ta postać to badacz	Grzejnik jest jak badacz
72	Blizny to pamiętnik	Blizny są jak pamiętnik	Ten egzemplarz to pamiętnik	Młot jest jak pamiętnik
73	Rodzina to korzeń	Rodzina jest jak korzeń	Ten badyl to korzeń	Lodówka jest jak korzeń
74	Poród to bitwa	Poród jest jak bitwa	Ta demonstracja to bitwa	Wstążka to bitwa
75	Umysł to pracownia	Umysł jest jak pracownia	Ten pokój to pracownia	Kalafior jest jak pracownia
76	Podświadomość to anioł	Podświadomość jest jak anioł	Ta figura to anioł	Pisak to anioł
77	Wanna to basen	Wanna jest jak basen	Ten zbiornik to basen	Koc jest jak basen
78	Pulpit komputera to biurko	Pulpit komputera jest jak biurko	Ten staroć to biurko	Lalka jest jak biurko
79	Nienawiść to błoto	Nienawiść jest jak błoto	Ta kałuża to błoto	Ten baran to błoto
80	Jej grube włosy to czapka	Jej grube włosy są jak czapka	Ten upominek to czapka	Banan to czapka
81	Artysta to dowódca	Artysta jest jak dowódca	Jego dziadek to dowódca	Chusteczka jest jak dowódca
82	Ta technologia to dzieciak	Ta technologia jest jak dzieciak	Ten chłopiec to dzieciak	Pineska jest jak dzieciak
83	Samotność to dziura	Samotność jest jak dziura	Ta pułapka to dziura	Ta ciężarówka to dziura
84	Przyjaźń to imperium	Przyjaźń jest jak imperium	To mocarstwo to imperium	Ta zasłona to imperium
85	Parasol to kapelusz	Parasol jest jak kapelusz	Ta rzecz to kapelusz	Czekolada jest jak kapelusz
86	Dobry film to kawa	Dobry film jest jak kawa	Ten proszek to kawa	Opona jest jak kawa
87	Mądrość to kręgosłup	Mądrość jest jak kręgosłup	Te kości to kręgosłup	Ta patelnia to kręgosłup
88	Fryzjerka to królowa	Fryzjerka jest jak królowa	Ta pszczoła to królowa	Ta doniczka to królowa
89	Izolacja to kurtka	Izolacja jest jak kurtka	To odzienie to kurtka	Te łyżwy to kurtka
90	Ten poradnik to lekarstwo	Ten poradnik jest jak lekarstwo	Ten płyn to lekarstwo	Ta choinka to lekarstwo
91	Jego komentarz to lektura	Jego komentarz jest jak lektura	Ten dramat to lektura	Prąd jest jak lektura
92	Dzieciństwo to łąka	Dzieciństwo jest jak łąka	Ta przestrzeń to łąka	Firana to łąka
93	Drwina to morderca	Drwina jest jak morderca	Ten nastolatek to morderca	Pianka to morderca
94	Ten płaszcz to namiot	Ten płaszcz jest jak namiot	Ich baza to namiot	Ten balet jest jak namiot
95	Dziecko to obrazek	Dziecko jest jak obrazek	To dzieło to obrazek	Dziurkacz to obrazek
96	Pieniądze to paliwo	Pieniądze są jak paliwo	Ta substancja to paliwo	Ten dokument to paliwo
97	Ta uwaga to pistolet	Ta uwaga jest jak pistolet	Ten eksponat to pistolet	Bombka to pistolet
98	Jej bagażnik to piwnica	Jej bagażnik jest jak piwnica	To schronienie to piwnica	Ta smycz jest jak piwnica
99	Jej szept to poduszka	Jej szept jest jak poduszka	To podparcie to poduszka	Ołówek jest jak poduszka
100	Telefon komórkowy to portfel	Telefon komórkowy jest jak portfel	Ta niespodzianka to portfel	Śnieg jest jak portfel
101	Budzik to porucznik	Budzik jest jak porucznik	Ten obywatel porucznik	Teczka to porucznik
102	Encyklopedia to posiłek	Encyklopedia jest jak posiłek	Ta nagroda to posiłek	Ten autobus to posiłek
103	Etyka to prawnik	Etyka jest jak prawnik	Ta kobieta to prawnik	Ten renifer to prawnik
104	Studia to przedszkole	Studia są jak przedszkole	Ten gmach to przedszkole	Ta herbata jest jak przedszkole
105	Internet to przychodnia	Internet jest jak przychodnia	Ta kamienica to przychodnia	Ta opaska jest jak przychodnia
106	Ta konferencja to przystanek	Ta konferencja jest jak przystanek	Ten słup to przystanek	Kolczyk to przystanek
107	Ten kraj to puszka	Ten kraj jest jak puszka	Ten pojemnik to puszka	Ta maskotka to puszka
108	Motywacja to silnik	Motywacja jest jak silnik	To urządzenie to silnik	Ten sweter jest jak silnik
109	Narty to skrzydła	Narty są jak skrzydła	Te elementy to skrzydła	Wizytówki to skrzydła
110	Humanista to słownik	Humanista jest jak słownik	Ten tom to słownik	Ta skarpetka jest jak słownik
111	Małżeństwo to spacer	Małżeństwo jest jak spacer	Ta czynność to spacer	Konewka jest jak spacer
112	Jego dłonie to stolik	Jego dłonie są jak stolik	Ten antyk to stolik	Ocean to stolik
113	Planeta to talerz	Planeta jest jak talerz	To znalezisko archeologiczne to talerz	Piłkarz jest jak talerz
114	Absolutorium to wesele	Absolutorium jest jak wesele	Ta impreza to wesele	Szczotka jest jak wesele
115	Autorytet to wieża	Autorytet jest jak wieża	Ten maszt to wieża	Ten notatnik to wieża
116	Kłótnia to wybuch	Kłótnia jest jak wybuch	Ten hałas to wybuch	Ta siatka jest jak wybuch
117	Ta powieść to wykład	Ta powieść jest jak wykład	Te zajęcia to wykład	Kapitan to wykład
118	Stypendium to wzgórze	Stypendium jest jak wzgórze	Ten pejzaż to wzgórze	Pociąg to wzgórze
119	Ten egzamin to zamach	Ten egzamin jest jak zamach	To wydarzenie to zamach	Krem to zamach
120	Komputer to zeszyt	Komputer jest jak zeszyt	Ten kalendarz to zeszyt	Trampki są jak zeszyt

## Appendix 2

See Table 10.

**Table 10:** English stimuli

	Novel nominal metaphors	Novel similes	Literal sentences	Anomalous sentences
1	A house is a harbour	A house is like a harbour	This area is a harbour	This banana is like a harbour
2	The past is an anchor	The past is like an anchor	This chain is an anchor	A shampoo is an anchor
3	Motivation is an engine	Motivation is like an engine	This machine is an engine	A frog is like an engine
4	Hope is a candle	Hope is like a candle	This wax is a candle	A cactus is a candle
5	Conversations are bridges	Conversations are like bridges	These connections are bridges	Cherries are like bridges
6	My head is a basket	My head is like a basket	This box is a basket	Champagne is a basket
7	Phobia is an enemy	Phobia is like an enemy	This politician is an enemy	A tomato is like an enemy
8	My mother is an elephant	My mother is like an elephant	This animal is an elephant	A dress is an elephant
9	My cat is a singer	My cat is like a singer	This lady is a singer	A broccoli is like a singer
10	Her words are jewellery	Her words are like jewellery	These stones are jewellery	A storm is jewellery
11	My lover is candy	My lover is like candy	This snack is candy	A doormat is like candy
12	Disability is a label	Disability is like a label	This piece of paper is a label	A garage is a label
13	A lie is a curtain	A lie is like a curtain	This material is a curtain	A sausage is like a curtain
14	A smile is a sweetener	A smile is like a sweetener	This caramel is a sweetener	A shark is a sweetener
15	His voice is a lullaby	His voice is like a lullaby	This song is a lullaby	This tiger is like a lullaby
16	Her neck is a tower	Her neck is like a tower	This pile is a tower	A pigeon is like a tower
17	A migraine is a kidnapper	A migraine is like a kidnapper	This teenager is a kidnapper	That potato is a kidnapper
18	Failures are bruises	Failures are like bruises	These marks are bruises	Bottles are like bruises
19	A fridge is a pocket	A fridge is like a pocket	This hole is a pocket	A vitamin is a pocket
20	Praise is a reward	Praise is like a reward	This medal is a reward	A finger is like a reward
21	Details are buttons	Details are like buttons	These fasteners are buttons	These knives are buttons
22	Religion is a watcher	Religion is like a watcher	An observer is a watcher	A cabbage is like a watcher
23	This company is a toddler	This company is like a toddler	My sister is a toddler	Pasta is a toddler
24	His decision is a bullet	His decision is like a bullet	This metal projectile is a bullet	Dust is like a bullet
25	Truth is a washer	Truth is like a washer	This facecloth is a washer	Cheese is a washer
26	This lecture is a prayer	This lecture is like a prayer	My wish is a prayer	A mushroom is like a prayer
27	Loneliness is a cavity	Loneliness is like a cavity	A crater is a cavity	A postcard is a cavity
28	Apologies are stitches	Apologies are like stitches	These threads are stitches	Airports are like stitches
29	Music is sugar	Music is like sugar	This sweetener is sugar	An elbow is sugar
30	Support is a charger	Support is like a charger	This device is a charger	Her towel is like a charger
31	Skyscrapers are chimneys	Skyscrapers are like chimneys	These ventilators are chimneys	Gingerbreads are chimneys
32	A kiss is honey	A kiss is like honey	This syrup is honey	A curtain is like honey
33	Protection is a blanket	Protection is like a blanket	This cloth is a blanket	Chocolate is a blanket
34	Pessimism is an acid	Pessimism is like an acid	This substance is an acid	A station is like an acid
35	My wardrobe is a temple	My wardrobe is like a temple	A holy place is a temple	That dolphin is a temple
36	A pet store is an orphanage	A pet store is like an orphanage	This institution is an orphanage	Rice is like an orphanage
37	These classrooms are gardens	These classrooms are like gardens	These grounds are gardens	Salmons are gardens
38	Success is a summit	Success is like a summit	This peak is a summit	This magazine is like a summit
39	University is a factory	University is like a factory	This establishment is a factory	This duck is a factory
40	Psychotherapies are surgeries	Psychotherapies are like surgeries	These medical procedures are surgeries	Peaches are like surgeries
41	Alcoholics are prisoners	Alcoholics are like prisoners	These villains are prisoners	These suitcases are prisoners
42	Ambition is a mountain	Ambition is like a mountain	This hill is a mountain	A butcher is a mountain
43	Achievement is a ceiling	Achievement is like a ceiling	This surface is a ceiling	A diary is a ceiling
44	God is an accuser	God is like an accuser	This man is an accuser	An iron is like an accuser
45	Legends are whispers	Legends are like whispers	These murmurs are whispers	Watermelons are whispers
46	Language is a currency	Language is like a currency	This cash is a currency	Rats are a currency
47	Bars are churches	Bars are like churches	These monuments are churches	Lips are like churches
48	A shopaholic is a hunter	A shopaholic is like a hunter	His brother is a hunter	A glove is a hunter
49	Cafes are offices	Cafes are like offices	These studios are offices	Kangaroos are like offices
50	This feminist is a lioness	This feminist is like a lioness	This creature is a lioness	A vase is a lioness
51	Skills are tickets	Skills are like tickets	These items are tickets	Giraffes are like tickets
52	Trees are fingers	Trees are like fingers	These bones are fingers	Doors are fingers
53	The Internet is a chamber	The Internet is like a chamber	This corridor is a chamber	A strawberry is like a chamber
54	A mosquito is a refugee	A mosquito is like a refugee	This rebel is a refugee	Coffee is a refugee
55	Eyes are windows	Eyes are like windows	These screens are windows	These pigs are like windows
56	This lake is a mirror	This lake is like a mirror	This glass is a mirror	This breakfast is a mirror
57	My thoughts are a liquid	My thoughts are like a liquid	This fluid is a liquid	A cheek is like a liquid
58	Her hairdo is a sculpture	Her hairdo is like a sculpture	This wood is a sculpture	A sheet is a sculpture
59	Knowledge is luggage	Knowledge is like luggage	These belongings are luggage	Hail is like luggage
60	Acrobats are spiders	Acrobats are like spiders	These insects are spiders	Cows are spiders
61	Bacteria are fighters	Bacteria are like fighters	These boxers are fighters	Gifts are like fighters
62	Government is a parent	Government is like a parent	My father is a parent	A tram is a parent
63	Conflicts are scissors	Conflicts are like scissors	These instruments are scissors	Plums are like scissors
64	Grandparents are libraries	Grandparents are like libraries	These book collections are libraries	Knees are libraries
65	A dream is a river	A dream is like a river	This stream is a river	An owl is like a river
66	This battlefield is a funeral	This battlefield is like a funeral	This celebration is a funeral	A monkey is a funeral
67	Adoption is a wedding	Adoption is like a wedding	This ceremony is a wedding	This conditioner is like a wedding
68	Forgiveness is a cookie	Forgiveness is like a cookie	A tasty thing is a cookie	A couch is a cookie
69	A hug is a jacket	A hug is like a jacket	This piece of clothing is a jacket	A beetroot is like a jacket
70	Viruses are travellers	Viruses are like travellers	These people are travellers	Tables are travellers
71	That boat is a coffin	That boat is like a coffin	This casket is a coffin	A lunch is like a coffin
72	Security is a chapel	Security is like a chapel	This sanctuary is a chapel	This camel is a chapel
73	Loans are cages	Loans are like cages	These enclosures are cages	Flowers are like cages
74	A bedroom is a kingdom	A bedroom is like a kingdom	This country is a kingdom	A paperclip is a kingdom
75	The brain is a container	The brain is like a container	This bucket is a container	Forks are like a container
76	Guns are judges	Guns are like judges	His cousins are judges	Earphones are like judges
77	My heart is a drawer	My heart is like a drawer	This piece of furniture is a drawer	A bug is a drawer
78	Poetry is cotton	Poetry is like cotton	This fiber is cotton	A peacock is like cotton
79	Her soul is a boulder	Her soul is like a boulder	This rock is a boulder	A napkin is a boulder
80	Networks are forests	Networks are like forests	These plantations are forests	Ties are like forests
81	My room is a castle	My room is like a castle	This big building is a castle	A dot is a castle
82	Determination is a ladder	Determination is like a ladder	This equipment is a ladder	A bee is like a ladder
83	A photograph is a diary	A photograph is like a diary	This blog is a diary	A hamster is a diary
84	Businessmen are wallets	Businessmen are like wallets	These purses are wallets	These cakes are like wallets
85	Calmness is an angel	Calmness is like an angel	This figure is an angel	Mold is an angel
86	Beliefs are anthems	Beliefs are like anthems	These melodies are anthems	These trousers are like anthems
87	Lifetime is a movie	Lifetime is like a movie	This drama is a movie	Snow is a movie
88	Problems are wrinkles	Problems are like wrinkles	These lines are wrinkles	Blankets are like wrinkles
89	Criticism is a freezer	Criticism is like a freezer	This cooler is a freezer	A pineapple is a freezer
90	Prejudice is a poison	Prejudice is like a poison	This liquid is a poison	A planet is like a poison
91	Memory is a muscle	Memory is like a muscle	This tissue is a muscle	This cauliflower is a muscle
92	This car is an oven	This car is like an oven	This appliance is an oven	A notebook is like an oven
93	Peace is a healer	Peace is like a healer	This person is a healer	This handbag is a healer
94	Children are parrots	Children are like parrots	These birds are parrots	Lollipops are like parrots
95	Alcohol is the devil	Alcohol is like the devil	This supernatural entity is the devil	A leaf is the devil
96	Tears are a lesson	Tears are like a lesson	This seminar is a lesson	A fox is like a lesson
97	Critics are razors	Critics are like razors	These blades are razors	Chickens are razors
98	The ocean is a carpet	The ocean is like a carpet	That covering is a carpet	A butterfly is like a carpet
99	The skull is a helmet	The skull is like a helmet	This safety hat is a helmet	A sofa is like a helmet
100	Laziness is a rebel	Laziness is like a rebel	That soldier is a rebel	This flowerpot is like a rebel
101	Disease cells are burglars	Disease cells are like burglars	These men are burglars	These socks are burglars
102	Conscience is a preacher	Conscience is like a preacher	That individual is a preacher	A blueberry is like a preacher
103	Experience is an adviser	Experience is like an adviser	A psychologist is an adviser	A bus is an adviser
104	The gas tank is a stomach	The gas tank is like a stomach	This organ is a stomach	This horse is like a stomach
105	His dog is a scientist	His dog is like a scientist	Her uncle is a scientist	A pumpkin is a scientist
106	His letters are sermons	His letters are like sermons	These talks are sermons	Spoons are sermons
107	A stalker is a shadow	A stalker is like a shadow	This shaded part is a shadow	Jogging is like a shadow
108	Sarcasm is a weapon	Sarcasm is like a weapon	A knife is a weapon	Fridge is a weapon
109	Medicines are wrestlers	Medicines are like wrestlers	These sportsmen are wrestlers	These carrots are like wrestlers
110	Vaccines are umbrellas	Vaccines are like umbrellas	These gadgets are umbrellas	These commercials are umbrellas
111	This ballad is a pillow	This ballad is like a pillow	A cushion is a pillow	A fence is like a pillow
112	A model is a needle	A model is like a needle	This object is a needle	The sky is a needle
113	His approval is a salary	His approval is like a salary	This payment is a salary	A cockroach is like a salary
114	This corporation is an onion	This corporation is like an onion	This ingredient is an onion	This canary is an onion
115	Friendship is a shelter	Friendship is like a shelter	This cave is a shelter	An ant is like a shelter
116	Earnings are badges	Earnings are like badges	These trademarks are badges	Ladybirds are badges
117	Chromosomes are siblings	Chromosomes are like siblings	These girls are siblings	Bikes are like siblings
118	Imagination is an inventor	Imagination is like an inventor	This scholar is an inventor	This balloon is an inventor
119	Grudge is a tumour	Grudge is like a tumour	This growth is a tumour	A daffodil is like a tumour
120	Rumours are runners	Rumours are like runners	These athletes are runners	Suits are runners
